# Psychopathology and Theory of Mind in patients with personality disorders

**DOI:** 10.1186/s40479-023-00224-1

**Published:** 2023-06-01

**Authors:** Juliane Burghardt, Silvia Gradl, Magdalena Knopp, Manuel Sprung

**Affiliations:** 1 Division of Clinical Psychology, Department of Psychology and Psychodynamics, Karl Landsteiner University of Health Sciences, Dr.-Karl-Dorrek-Straße 30, Krems an Der Donau, 3500 Austria; 2 University Hospital for Psychosomatic Medicine Eggenburg, Grafenberger Straße 2, Eggenburg, 3730 Austria; 3 Faculty of Psychology and Educational Sciences, Department of Psychology, Ludwig-Maximilians-Universität München, Leopoldstraße 13, Munich, 80802 Germany

**Keywords:** Borderline personality disorder, Theory of Mind, Depression, Mixed personality disorders, Symptom severity

## Abstract

**Objective:**

People with mental disorders frequently suffer from deficits in the ability to infer other’s mental states (Theory of Mind; ToM). Individuals with borderline personality disorder (BPD) show ToM deficits characterized by exceeding ToM (over-attributions of mental states). The present study analyzed associations between ToM, BPD severity, and depression severity in patients with BPD and other personality disorders.

**Method:**

We analyzed ToM abilities in 128 patients with BPD and 82 patients with ‘mixed and other personality disorders’ (MOPD). MOPD are diagnosed if symptoms of multiple personality disorders are present without any set of symptoms being dominant enough to allow a specific diagnosis. We used the movies for the assessment of social cognition (MASC) to measure ToM abilities, the Patient Health Questionnaire (PHQ-9) to assess severity of depression and the McLean Screening Instrument for Borderline Personality Disorder (MSI-BPD) to assess the severity of BPD symptoms.

**Results:**

Both symptoms of BPD and depression were associated with exceeding ToM in separate regressions. Using a stepwise regression, only the association of depression severity with exceeding ToM was reliable. Patients with BPD and MOPD did not differ in exceeding ToM. Age was most reliably associated with ToM.

**Conclusion:**

The results imply that exceeding ToM is associated with general psychopathology instead of BPD-symptoms in specific. Patients with MOPD show deficits similar to BPD patients.

## Introduction

The Theory of Mind (ToM) refers to the ability to ascribe internal states, such as desires, beliefs, intentions, and emotions, to others and to explain and predict behavior on the basis of these mental states [13, 37]. A recent review of meta-analyses supported the association between mental disorders and ToM impairments by showing ToM deficits across 30 clinical conditions [13]. The Research Domain Criteria framework (RDoc; [35, 36]), which seeks to describe mental disorders in terms of their underlying impairment rather than by distinct categorical diagnoses, suggested ToM (i.e., understanding mental states of others) as a likely transdiagnostic factor by which mental health should be described.

ToM is part of a wide array of social cognition processes, which refer to the mental operations that underlie social interactions [13]. Social cognition refers to the processes by which humans perceive, interpret, and respond to social stimuli within their specific context [3, 11, 14]. Understanding other’s intentions, beliefs, and emotions (i.e., ToM) is crucial for socially adequate responses [32, 50] and successful social functioning [46]. Hence, ToM impairments can result in poor social adjustment [1].

Irrespective of the substantial evidence for ToM deficits in mental disorders (e.g., [13]), evidence regarding ToM among patients with personality disorders is relatively limited with the exception of borderline personality disorder (BPD). BPD is characterized by difficulties in social and interpersonal interactions [2, 29]. There is ample evidence that people with BPD suffer from ToM impairments compared to healthy controls [9, 19, 37, 44]. Importantly, ToM impairments can take on different forms; ToM deficits in BPD patients are typically marked by a tendency to ascribe extreme mental states to others [49]. This is often referred to as exceeding ToM or hypermentalising. This tendency involves drawing inferences from behavior that others would not find justified. In contrast, other disorders are more typically associated with reduced ToM (for instance alcohol use disorder; [41]), which implies a lower chance to ascribed mental states to others.

A recent meta-analysis challenged the idea that exceeding ToM was specific to BPD [34]. The meta-analysis concluded that exceeding ToM was found in a broad range of mental disorders (e.g., schizophrenia, autism spectrum disorder and persistent somatoform pain disorder). This meta-analysis reviewed associations between psychopathology and exceeding ToM and concluded that the association between exceeding ToM and psychopathology did not differ between BPD and other disorders. Thus, exceeding ToM was associated with general psychopathology. However, the applicability of this conclusion to personality disorders is limited, as only one study with a personality disorder other than BPD was included. This study did not find evidence for a significant association between psychopathology and exceeding ToM among individuals with antisocial personality disorder ([38] see [34]).

Further, McLaren and colleagues [34] only reviewed psychopathology directly related to the diagnosis, for instance they analyzed the effect of BPD-symptom severity in BPD patients. However, other symptoms could also affect ToM; only few studies have explored this question. There are, however, studies related to comorbidity of BPD and depression. These studies showed mixed results. A previous meta-analysis found that patients with major depressive disorder (MDD) had more ToM deficits than patients with BPD alone, patients with BPD and comorbid MDD actually had a better ToM than those who solely suffered from BPD or MDD [44]. In contrast, a later study could not replicate this finding. Instead, results showed that BPD patients with MDD had lower ToM abilities than BPD patients without MDD [57]. Thus, findings regarding the effect of comorbid depression on ToM abilities in BPD are contradictory.

A recent study by Normann-Eide and colleagues [40] found that severity of personality psychopathology and general severity of symptom distress were associated with exceeding ToM, in both patients with BPD and other personality disorders (OPD). Thus, the severity of psychopathology was associated to exceeding ToM in all patients and not limited to BPD patients. The study concluded that, general symptom distress might be more relevant to exceeding ToM than specific symptoms of personality disorders.

The interpretation of the ToM literature is complicated by the fact that many studies use the Reading the Mind in the Eyes Test (RMET; [6]) to measure ToM. This test does not fulfill all criteria for a ToM test and should therefore not be used to assess ToM. ToM measures require participants to represent other’s mental states as well as to distinguish between their own and the other’s mental state [43]. A test that fulfills these criteria is the Movies for the Assessment of Social Cognition (MASC; [15]), which, measures more complex ToM abilities than the RMET [43]. The MASC is considered to be a relatively ecological valid task [34], it presents videos of social interactions close to real life encounters. Further, the task assesses two different ToM aspects, that is the affective and the cognitive ToM [33, 57]. Cognitive ToM involves representing thoughts, intentions, or beliefs. Affective ToM involves representing feelings. The MASC includes items about both affective and cognitive mental states, which assures a more complete ToM assessment.

The current study investigated ToM abilities in patients with personality disorders using the ecologically valid MASC. First, we tested whether exceeding ToM errors were also prevalent in personality disorders other than BPD. Second, in line with McLaren and colleagues [34] we expected that exceeding ToM would be associated with psychopathology. Third, we explored whether BPD-symptom severity or depression severity were associated to ToM. Previous evidence suggested that ToM abilities are influenced by biological sex and age; healthy women show better ToM performance than men [4, 5] and ToM performance declines with increasing age among healthy adults [20]. We therefore controlled for sex and age. To strengthen the validity of the results we used a big and thus, highly powered sample.

## Materials and methods

### Participants and design

The study analyzed data from 210 patients (158 women, 52 men) from an inpatient treatment unit for personality disorders. Patients’ age ranged from 19 to 61 years, with an average age of 33.56 years (SD = 10.42). The sample included 128 patients treated for BPD (F60.3) and 82 patients treated for mixed and other personality disorders (MOPD; F61). The MOPD diagnosis was based on the International Classification of Diseases 10 (ICD-10) [55]. A mixed personality disorder is diagnosed if symptoms of multiple specific personality disorders according to F60 are present but no set of symptoms is dominant enough to allow a specific diagnosis [55].

Table 1 compares demographic characteristics of the two patient groups. There were more women among patients diagnosed with BPD than among patients diagnosed with MOPD. Unsurprisingly, patients diagnosed with BDP reported more symptoms of BPD than patients diagnosed with MOPD. BPD patients were younger and had a lower education level than MOPD patients. The BPD and MOPD patients were comparable regarding marital status and symptoms of depression.

**Table 1:** Sociodemographic and clinical variables of patients diagnosed with BPD and MOPD

	**BPD diagnosis** *N* = 128 ***N* (%)**	**MOPD diagnosis** *N* = 82 ***N* (%)**	**Χ**^**2**^** (df)**	***p***
Sex			41.66 (1)	< .001
Men	12 (9.4)	40 (48.8)		
Women	116 (90.6)	42 (51.2)		
Education level			9.39 (1)	.002
Low education	88 (68.8)	39 (47.6)		
Medium / high education	40 (25.0)	43 (37.8)		
Marital status	128 (61.0)	82 (39.1)	5.66 (2)	.912
Single	81 (63.3)	52 (63.4)		
Married / in a relationship	35 (27.3)	21 (25.6)		
Divorced / separated / widowed	12 (9.4)	9 (11.0)		
Comorbidities^a^				
Neurotic, stress-related and somatoform disorders (F40-F48)	39.8%	30.5%		
Mood disorders (F30-F39)	18.8%	24.4%		
Eating disorders (F50)	13.3%	7.3%		
Hyperkinetic disorders (F90)	10.2%	4.9%		
Disorders of adult personality and behavior (F60-F69;	7.8%	8.5%		
	***M (SD)***	***M (SD)***	***t* (df)**	***p***
Age	30.30 (8.86)	38.66 (10.68)	6.16 (208)	< .001
Depression (PHQ-9)	16.37 (5.76)	15.09 (5.32)	-1.62 (208)	.107
BPD symptoms (MSI-BPD)	7.54 (2.38)^d^	6.35 (2.43)^e^	-3.54 (205)	< .001

*BPD* Borderline personality disorder, *MOPD* Mixed and other personality disorders

^a^Sorted by frequency in BPD group

^b^21 missing values

^c^4 missing values

^d^2 missing values

^e^1 missing values

Comorbidities were common among both patient groups. Among patients diagnosed with BPD 70.1% received at least one secondary diagnosis. Among patients diagnosed with MOPD 65.9% received at least one secondary diagnosis. Some patients received up to four secondary diagnoses. The frequency of common secondary diagnoses is described in Table 1. The most frequent secondary diagnoses were neurotic, stress-related and somatoform disorders (F40-F48; 39.8% among patients diagnosed with BPD and 30.5% among patients diagnosed with MOPD). This includes posttraumatic stress disorders, dissociative disorders, anxiety disorders and obsessive–compulsive disorders. The second most common comorbidity for patients diagnosed with BPD (18.8%) and for patients diagnosed with MOPD (24.4%) were mood disorders (F30-F39), which includes for example major depressive disorders and persistent mood disorders. The third and fourth most frequent diagnoses among patients diagnosed with BPD were eating disorders (F50; 13.3%) and hyperkinetic disorders (F90; 10.8%), which refers mainly to attention deficit hyperactivity disorder (ADHD). Among patients diagnosed with MOPD the third most frequent diagnoses were disorders of adult personality and behavior (F60-F69; 8.5%).

A post hoc power analysis with G*Power [16] confirmed the high power of the sample. The conducted regressions with 5 predictors had a power of > 0.99 to find a medium effect of f^2^ = 0.23 (equals Cohen’s d = 0.46) if it existed. The medium effects size reflects the estimated effect size for MASC studies previously reported in a meta-analysis of ToM deficits in BPD patients [37].

The study analyzed data that was collected as part of the routine examination at a university hospital for psychosomatic medicine in Austria. All patients of the specialized treatment unit were eligible if they were eighteen or older. The clinic treats patients that are able to participate in talk therapies. This means they needed to have at least conversational skills in German. Patients with acute psychotic symptoms, suicidal behavior, or acute intoxication are not admitted (for more details see [24]). Patients are typically referred to treatment by psychiatrists or other medical professionals. The hospitalization lasts between 8 to 12 weeks, during which the patients receive intense and disorder-specific therapy by an interdisciplinary team.

### Procedure

The data was collected between July 2017 and August 2020 as part of the routine inpatient clinical care in a psychiatric-psychosomatic hospital. Only data from the assessment at intake were analyzed. Basic sociodemographic data, such as age and sex, were obtained from the hospital information system. Symptoms of BPD, depression and ToM were measured using the Computer-based Health Evaluation System [21]. Patients answered the questionnaires in a computer assessment room with up to seven other patients in separated cubicles. The assessment at intake took 2 h, divided in two 1-h sessions. The psychiatric diagnosis was determined within an unstandardized clinical interview.

### Assessment

#### BPD symptoms

BPD symptoms were measured with the German version of the McLean Screening Instrument for Borderline Personality Disorder (MSI-BPD). It’s a 10-item measure that assesses symptoms of borderline personality disorder (e.g., “Have any of your closest relationships been troubled by a lot of arguments and repeated breakups?”, “Have you been extremely moody?”, “Have you deliberately hurt yourself physically?”, [58]). Answers are given in a yes /no format. The number of yes answers represents the borderline symptom score. It can range from 0 to 10. Higher scores indicate stronger BPD symptoms. Previous research has suggested that a useful clinical cutoff score to predict BPD among adults is 7 or higher [42]. The MSI-BPD previously showed good psychometric properties, with good internal consistency (α = 0.74), test-retest reliability (r_s_ = 0.72), good validity and good diagnostic accuracy [10, 39, 42, 58]. The German version performed equally well [27, 28].

#### Theory of Mind performance

The Movie for the assessment of social cognition (MASC) was used to assess ToM [15]. The MASC uses a 15-min movie divided into 45 short video clips showing social interactions between four actors (two women, two men) getting together for a dinner party. After each video clip the program asks participants about the characters’ feelings/emotions or thoughts/intentions. Questions appear on the screen together with four alternative answers. Each answer represents one of four response types: 1. Correct answer (i.e., accurate identification of characters’ feeling, emotion, thought, or intention), 2. Exceeding ToM (i.e., over-interpretative ToM or overmentalization), 3. Reduced ToM (i.e., insufficient ToM or undermentalization) and 4. No of ToM (i.e., complete lack of ToM or literal understanding). A total MASC score (max. score = 45) was calculated based on the number of correct answers. The number of each type of error, exceeding ToM, reduced ToM, and no ToM, was calculated. Beyond the 45 test questions, the MASC also includes six control questions related to physical events, with a respective set of answers representative of non-social inferences. The MASC has previously shown high reliability, internal consistency (α ≥ 0.82 for all scores), good test-retest stability and good convergent and discriminant validity [15, 17, 45].

#### Depression

Symptoms of depression were assessed with the German version of the depression module of the Patient Health Questionnaire (PHQ-9; [25]). Nine items related to DSM-IV criteria assessed the severity of depression symptoms (e.g., “Little interest or pleasure in doing things”,“Felling down, depressed, or hopeless”; “Feeling tired or having little energy”). Participants rated how often they were bothered by each symptom within the last 2 weeks on a scale from 0 (not at all) to 3 (nearly every day). Ratings are summed to a total severity score ranging from 0 to 27. Major depressive disorder (MDD) should be considered in patients who endorse ≥ 5 of the 9 symptoms as present “more than half the days” (the 9th item, “Thoughts that you would be better off dead or of hurting yourself in some way”, counts if endorsed as present on “several days”) and one of the first two symptoms (depressed mood or loss of interest) is endorsed. The PHQ-9 previously showed good psychometric properties, with high internal consistency (α = 0.86) and good test–retest reliability (*r* = 0.84), good convergent and discriminant validity, and good sensitivity to change [7, 26, 30, 30, 31, 31]. The German version of the PHQ-9 showed similarly good psychometric properties [18].

#### Diagnoses

The psychiatric diagnosis refers to the main diagnoses, which was given by a psychiatrist (coded with “1” for BPD diagnosis and “0” for MOPD) as part of the standard clinical routine. The diagnosis was based on ICD-10 and was derived within a clinical interview upon intake by integrating written and oral symptom reports by patients, and information about their medical history. None of the questionnaires described above were used to give diagnoses.

#### Sociodemographic data

Sex, age, and education level were derived from the hospital information system. Sex (male, female) is defined in terms of self-reported biological sex. Age refers to the chronological age (in years) at the time of admission. Education level was classified into two categories: “1” for no school-leaving qualification or compulsory school, “2” for secondary school and college degrees.

### Statistical analysis

First, we compared demographic variables, symptoms, and ToM between BPD and MOPD patients using Χ^2^ or t-tests. Afterwards, we conducted four sets of linear regressions. Each set of regressions used one of four (continuous) ToM outcomes as dependent variables. These four ToM outcomes were overall ToM performance and the frequencies of one of the three error types (exceeding ToM, reduced ToM, no ToM). The first set of regressions tested the association of ToM with BPD severity, while controlling for age, sex, and psychiatric diagnosis (BPD vs. MOPD). Thus, BPD severity, psychiatric diagnosis (BPD vs. MOPD), age, and sex were simultaneously entered as predictors for each of the four ToM outcomes. The second set of regressions exchanged BPD severity with depression severity and was otherwise identical. The third set of regressions entered both BPD severity and depression severity, as well as the control variables psychiatric diagnosis (BPD vs. MOPD), age, and sex. Since this set did not reach a conclusive result, we repeated the third set of regressions with stepwise inclusions of the same predictors. All analyses were computed using IBM SPSS (Version 27).

## Results

Table 2 presents the unadjusted differences in MASC performance between BPD and MOPD patients. BPD patients showed an overall better MASC performance than MOPD patients. This difference resulted from BPD patients making fewer reduced ToM errors.

**Table 2:** ToM in patients diagnosed with BPD and MOPD in the “Movie for the Assessment of Social Cognition” (MASC)

	**BPD diagnosis** ***M (SD)***	**MOPD diagnosis** ***M (SD)***	***F* (df)**	***P***	**η**_**p**_^**2**^
MASC total correct	31.41 (5.64)	29.54 (6.54)	4.84 (1, 208)	.029	0.023
MASC exceeding ToM	7.52 (3.75)	7.23 (3.45)	0.30 (1, 208)	.582	0.001
MASC reduced ToM	6.04 (2.91)	6.99 (2.90)	5.34 (1, 208)	.022	0.025
MASC no ToM	2.94 (2.03)	3.27 (2.65)	1.04 (1, 208)	.309	0.005

*BPD* Borderline personality disorder, *MOPD* Mixed and other personality disorders, *ToM* Theory of Mind

Regression results are presented in Tables 3 and 4. The first set of regressions (Regression 1, Table 3 and 4) tested associations between BPD-symptom severity and ToM. The regression showed that the severity of BPD was associated with exceeding ToM, while psychiatric diagnosis (MOPD and BPD) showed no such association.

**Table 3:** Regressions of diagnosis, BPD severity, depression severity, sex, and age on ToM total correct responses and exceeding ToM errors

	**ToM total**			**exceeding ToM**		
**Predictors**	**β**	***B***	**CI 95%**	**β**	***B***	**CI 95%**
**Regression 1 (enter)**						
**Sex ** (0 = female, 1 = male)	-0.03	-0.37	[-2.49, 1.74]	**0.16**	**1.31**	**[0.00, 2.62]**
**Age in years**	**-0.23**	**-0.13**	**[-0.22, -0.05]**	0.00	0.00	[-0.05, 0.05]
**BPD severity**	-0.10	-0.25	[-0.59, 0.08]	**0.16**	**0.23**	**[0.02, 0.44]**
**BPD vs. MOPD diagnosis**	0.10	1.18	[-0.74, 3.11]	0.06	0.43	[-0.76, 1.61]
**Regression 2 (enter)**						
**Sex ** (0 = female, 1 = male)	-0.02	-0.22	[-2.35, 1.90]	**0.17**	**1.39**	**[0.09, 2.68]**
**Age in years**	**-0.21**	**-0.12**	**[-0.21, -0.04]**	-0.01	-0.00	[-0.06, 0.05]
**Depression severity**	**-0.14**	**-0.15**	**[-0.29, -0.01]**	**0.18**	**0.12**	**[0.03, 0.21]**
**BPD vs. MOPD diagnosis**	0.08	0.95	[-0.96, 2.86]	0.09	0.65	[-0.51, 1.81]
**Regression 3 (enter)**						
**Sex ** (0 = female, 1 = male)	-0.02	-0.34	[-2.45, 1.77]	0.15	1.29	[-0.01, 2.59]
**Age in years**	-0.22	-0.12	[-0.21, -0.04]	-0.02	-0.01	[-0.06, 0.05]
**BPD severity**	-0.05	-0.13	[-0.49, 0.24]	0.10	0.15	[-0.07, 0.38]
**Depression severity**	-0.13	-0.14	[-0.29, 0.02]	0.13	0.08	[-0.01, 0.18]
**BPD vs. MOPD diagnosis**	0.10	1.28	[-0.64, 3.20]	0.05	0.37	[-0.82, 1.55]
**Regression 4 (stepwise)**						
**Age in years**	**-0.26**	**-0.15**	**[-0.23, -0.07]**	not included		
**Depression severity**	**-0.13**	**-0.14**	**[-0.28, -0.003]**	**0.17**	**0.11**	**[0.02, 0.20]**

Note. *BPD* borderline personality disorder, *MOPD* mixed and other personality disorders, *ToM* Theory of Mind, *CI 95%* 95% confidence interval; bold numbers are significant on a *p* = .05 level

**Table 4:** Regressions of diagnosis, BPD severity, depression severity, sex, and age on reduced and no ToM errors

	**reduced ToM**			**no ToM**		
**Predictors**	**β**	***B***	**CI 95%**	**β**	***B***	**CI 95%**
**Regression 1 (enter)**						
** Sex** (0 = female, 1 = male)	-0.06	-0.38	[-1.42, 0.67]	-0.10	-0.52	[-1.30, 0.26]
** Age in years**	**0.23**	**0.07**	**[0.02, 0.11]**	**0.18**	**0.04**	**[0.01, 0.07]**
** BPD severity**	-0.01	-0.01	[-0.17, 0.16]	0.01	0.01	[-0.11, 0.14]
** BPD vs. MOPD diagnosis**	-0.10	-0.62	[-1.57, 0.33]	-0.08	-0.37	[-1.08, 0.34]
**Regression 2 (enter)**						
** Sex** (0 = female, 1 = male)	-0.07	-0.46	[-1.50, 0.58]	-0.11	-0.61	[-1.44, 0.22]
** Age in years**	**0.22**	**0.06**	**[0.02, 0.10]**	**0.16**	**0.04**	**[0.00, 0.07]**
** Depression severity**	0.03	0.02	[-0.05, 0.09]	0.03	0.01	[-0.04, 0.07]
** BPD vs. MOPD diagnosis**	-0.11	-0.64	[-1.57, 0.30]	-0.06	-0.29	[-1.04, 0.45]
**Regression 3 (enter)**						
** Sex** (0 = female, 1 = male)	-0.06	-0.38	[-1.43, 0.66]	-0.10	-0.52	[-1.31, 0.26]
** Age in years**	**0.22**	**0.06**	**[0.02, 0.11]**	**0.18**	**0.04**	**[0.005, 0.07]**
** BPD severity**	-0.03	-0.04	[-0.22, 0.15]	-0.01	-0.01	[-0.15, 0.13]
** Depression severity**	0.06	0.03	[-0.05, 0.11]	0.06	0.02	[-0.04, 0.08]
** BPD vs. MOPD diagnosis**	-0.11	-0.64	[-1.59, 0.31]	-0.09	-0.38	[-1.09, 0.33]
**Regression 4 (stepwise)**						
** Age in years**	**0.25**	**0.07**	**[0.03, 0.11]**	**0.18**	**0.04**	**[0.01, 0.06]**

Note. *BPD* borderline personality disorder, *MOPD* mixed and other personality disorders, *ToM* Theory of Mind, *CI 95%* 95% confidence interval; bold numbers are significant on a *p* = .05 level

The second set of regressions (Regression 2, Tables 3 and 4) tested for effects of depression severity instead of BPD severity. Equal to BPD severity depression severity was associated with exceeding ToM. In addition, depression severity was associated with lower overall ToM performance. A Pearson correlation showed that BPD severity and depression severity were moderately correlated, *r* = 0.42.

The third set of regressions (Regression 3, Tables 3 and 4) tested BPD severity and depression severity simultaneously, which did not yield significant associations of either psychopathology with ToM. The last set of regressions instead tested the same predictors stepwise. In these analyses depression severity was associated with overall ToM and exceeding ToM.

Age affected the overall ToM, as well as reduced and no ToM errors; younger patients performed better than older patients. There was no significant effect of age on exceeding ToM. Men made more exceeding ToM errors than women. This effect was not reliable in the stepwise regression. Otherwise, sex did not affect results.

## Discussion

The present study found that ToM was associated with both the severity of symptoms of depression and symptoms of BPD in patients with personality disorders. This association was driven by exceeding ToM errors, which were more prevalent in patients with more severe psychopathology. Exceeding ToM implies that behaviors are over-interpreted in a way that ascribe intentions where others would not. The association between exceeding ToM and psychopathology was driven by the severity of depression and not BPD-symptom severity. Other ToM errors (i.e., reduced ToM and no ToM) showed no association with symptom severity. The psychiatric diagnosis of BPD or MOPD was not associated with exceeding ToM.

Previous research has established that BPD patients are likely to show exceeding ToM compared to healthy controls [19, 37]. Further, the results support the notion that exceeding ToM is associated with psychopathology in general [34]. The current study expands this association between psychopathology and exceeding to patients with the diagnosis of mixed and other personality disorders. Interestingly, the results show that depression severity is more reliably associated with exceeding ToM than severity of BPD symptoms. This supports the idea that general psychopathology is associated with exceeding ToM rather than BPD symptoms specifically. This further supports the transdiagnostic nature of ToM (RDoc; [35, 36]) and its deficits since it is less clearly related to symptoms specific to BDP.

The current results did not find differences between BPD and MOPD patients regarding exceeding ToM. In contrast, a previous study had found that BDP patients more frequently showed excessive ToM than patients with other personality disorders [40]. The study by Normann-Eide and colleagues [40] showed that when differences in severity of pathology and differences in criteria of personality disorders were accounted for, the diagnoses no longer predicted exceeding ToM. Similarly, the current study showed that BPD severity and depression severity were better predictors of ToM abilities than diagnoses. However, the current study did not account for severity of other personality disorders. Both studies relied on the MASC to measure ToM. A different study found that ToM deficits were specific to BPD relative to other personality disorders even when general symptom distress was controlled for [48]. This study relied on an interview using autobiographical memories to measure ToM. Interestingly, the meta-analysis by McLaren and colleagues [34] suggested that it is important to test whether exceeding ToM deficits would be more pronounced among BPD patients if the measure would have personal relevance and emotional salience, which are suspected to increase ToM deficits among BPD patients.

Our findings complement results on the association of depression and ToM. Symptoms of depression were associated with exceeding ToM and lower overall ToM performance. This is in line with a study by Zabihzadeh and colleguage [57], which found that BPD patients with MDD had lower ToM abilities than those without MDD. However, in contrast to our study previous studies had shown that depressed patients made more reduced ToM errors than HC [54], which we cannot test without a health control group. In contrast to our findings that showed increased depression symptoms among BPD patients were associated with a higher rate of exceeding ToM errors, a meta-analysis by Richman and Unoka [44] found that ToM performance increased among patients with a combination of BPD and depression. However, these findings relied on different measures; ToM results are influenced by the type of measure used [43].

The current findings suggest that patients with personality disorders and severe symptoms of depression are at a high risk of ToM impairment and might therefore have an increased need for ToM skills trainings. Multiple treatments of mental illness focus on the improvement of social cognition skills [23, 52]. They were efficient in both patients with depression [22] and BPD [47]. It should be tested whether these treatments are beneficial to patients with personality disorders and severe depression.

Earlier effects of age on ToM could be replicated [20]. ToM abilities were higher among younger patients than older patients. This is in line with a meta-analysis showing more ToM errors among older individuals [20]. Only exceeding ToM was not significantly related to age. In line with previous findings, which showed that women perform better than men on ToM measures [4, 5], men made more exceeding ToM errors then women. However, this association was not reliable in the stepwise regression and contradicts the findings of a previous meta-analysis on ToM in BPD patients, which did not find an effect of sex-ratio on ToM [37].

A limitation of the study is that it did not assess the severity of other personality disorders. This limits our ability to test the association with other personality disorders and exceeding ToM. The mixed and other personality disorders category combines a multitude of possible symptoms. Explicit coding of the different personality disorder criteria would have allowed more detailed analyses. Further, the current study used a short measure of BPD symptoms meant for screening [58]. Future studies should replicate this findings with more thorough measures of BPD symptoms such as the Borderline Symptom List (BSL; [8]). Another shortcoming of the current study is, that it analyzed psychiatric diagnoses based on the ICD-10 determined within the clinical routine and without a standardized structured clinical interview. While diagnoses in previous research are predominantly based on the Diagnostic and Statistical Manual of Mental Disorders (DSM) measured by the Structured Clinical Interview for DSM-IV Axis II (SCID-II; [37, 53]). However, ICD diagnoses are commonly used in Europe and mandatory for billing, further, they typically become the basis for treatment recommendations. Therefore, the finding that ICD-10 diagnoses asses within an unstandardized interview were not associated with ToM have high practical relevance. The finding supports the need for standardized measures in clinical care. Another limitation of the present study is the absence of healthy participants. It is unclear whether the association between BPD severity and ToM would generalize to the general population since BPD symptoms are much more prevalent in clinical samples [12]. Further, without a healthy control it remains unclear whether the BPD and MOPD groups show ToM deficits. However, descriptively patients diagnosed with BPD within the current sample performed worse on the MASC than BPD patients within previous samples [3, 40, 51]. This makes it likely that the current results indicate ToM deficits.

## Conclusion

In the current study, overall ToM performance was not found to differ between patients with BPD and MOPD when age and gender were considered. Exceeding ToM increased with both higher BPD-symptom severity and depression severity in individuals with BPD and MOPD diagnoses. Depression severity was more reliably associated with exceeding ToM than BPD-symptom severity. Thus, general psychopathology was a stronger predictor of ToM deficits than BPD-specific symptoms.

## Data Availability

The datasets presented in this article are not readily available because of the vulnerability of the study sample. Participants of this study did not agree for their data to be shared publicly, so supporting data is not available.
