# Correction: Psychopathology and theory of mind in patients with personality disorders

**DOI:** 10.1186/s40479-024-00260-5

**Published:** 2024-09-11

**Authors:** Juliane Burghardt, Silvia Gradl, Magdalena Knopp, Manuel Sprung

**Affiliations:** 1 Division of Clinical Psychology, Department of Psychology and Psychodynamics, Karl Landsteiner University of Health Sciences, Dr.-Karl-Dorrek-Straße 30, Krems an Der Donau, 3500 Austria; 2 University Hospital for Psychosomatic Medicine Eggenburg, Grafenberger Straße 2, Eggenburg, 3730 Austria; 3 Faculty of Psychology and Educational Sciences, Department of Psychology, Ludwig-Maximilians- Universität München, Leopoldstraße 13, 80802 Munich, Germany

**Borderline Personality Disorder and Emotion Dysregulation (2023) 10:18**

10.1186/s40479-023-00224-1.

Following publication of the original article [1], we have been notified that Tables 3 and 4 were published and aligned incorrectly.

Incorrect Tables 3 and 4 are shown below:

**Table 3:** Regressions of diagnosis, BPD severity, depression severity, sex, and age on ToM total correct responses and exceeding ToM errors

Predictors	ToM total						Exceeding ToM		
	β		*B*			CI 95%	β	*B*	CI 95%
**Regression 1 (enter)**									
**Sex** (0 = female, 1 = male)	–0.03		–0.37			[–2.49, 1.74]	**0.16**	**1.31**	**[0.00, 2.62]**
**Age in years**	**–0.23**		**–0.13**			**[–0.22, –0.05]**	0.00	0.00	[–0.05, 0.05]
**BPD severity**	–0.10		–0.25			[–0.59, 0.08]	**0.16**	**0.23**	**[0.02, 0.44]**
**BPD vs. MOPD diagnosis**	0.10		1.18			[–0.74, 3.11]	0.06	0.43	[–0.76, 1.61]
**Regression 2 (enter)**									
**Sex** (0 = female, 1 = male)	–0.02		–0.22			[–2.35, 1.90]	**0.17**	**1.39**	**[0.09, 2.68]**
**Age in years**	**–0.21**		**–0.12**			**[–0.21, –0.04]**	–0.01	–0.00	[–0.06, 0.05]
**Depression severity**	**–0.14**	**–0.15**	**[–0.29, –0.01]**	**0.18**	**0.12**	**[0.03, 0.21]**			
**BPD vs. MOPD diagnosis**	0.08	0.95	[–0.96, 2.86]	0.09	0.65	[–0.51, 1.81]			
**Regression 3 (enter)**									
**Sex** (0 = female, 1 = male)	–0.02		–0.34			[–2.45, 1.77]	0.15	1.29	[–0.01, 2.59]
**Age in years**	–0.22		–0.12			[–0.21, –0.04]	–0.02	–0.01	[–0.06, 0.05]
**BPD severity**	–0.05	–0.13	[–0.49, –0.24]	0.10	0.15	[–0.07, 0.38]			
**Depression severity**	–0.13	–0.14	[–0.29, –0.02]	0.13	0.08	[–0.01, 0.18]			
**BPD vs. MOPD diagnosis**	0.10	1.28	[–0.64, 3.20]	0.05	0.37	[–0.82, 1.55]			
**Regression 4 (stepwise)**									
**Age in years**	**–0.26**	**–0.15**	**[–0.23, –0.07]**	not included					
**Depression severity**	**–0.13**	**–0.14**	**[–0.28, –0.003]**	**0.17**	**0.11**	**[0.02, 0.20]**			

Bold numbers are significant on a *p* = .05 level

*BPD* Borderline personality disorder, *MOPD* Mixed and other personality disorders, *ToM* Theory of Mind, *CI 95%* 95% confidence interval

**Table 4:** Regressions of diagnosis, BPD severity, depression severity, sex, and age on reduced and no ToM errors

Predictors	Reduced ToM						No ToM		
	β		*B*			CI 95%	β	*B*	CI 95%
**Regression 1 (enter)**									
**Sex** (0 = female, 1 = male)	–0.06		–0.38			[–1.42, 0.67]	–0.10	–0.52	[–1.30, 0.26]
**Age in years**	**0.23**		**0.07**			**[0.02, 0.11]**	**0.18**	**0.04**	**[0.01, 0.07]**
**BPD severity**	–0.01		–0.01			[–0.17, 0.16]	0.01	0.01	[–0.11, 0.14]
**BPD vs. MOPD diagnosis**	–0.10		–0.62			[–1.57, 0.33]	–0.08	–0.37	[–1.08, 0.34]
**Regression 2 (enter)**									
**Sex** (0 = female, 1 = male)	–0.07		–0.46			[–1.50, 0.58]	–0.11	–0.61	[–1.44, 0.22]
**Age in years**	**0.22**		**0.06**			**[0.02, 0.10]**	**0.16**	**0.04**	**[0.00, 0.07]**
**Depression severity**	0.03	0.02	[–0.05, 0.09]	0.03	0.01	[–0.04, 0.07]			
**BPD vs. MOPD diagnosis**	–0.11	–0.64	[–1.57, 0.30]	–0.06	–0.29	[–1.04, 0.45]			
**Regression 3 (enter)**									
**Sex** (0 = female, 1 = male)	–0.06		–0.38			[–1.43, 0.66]	–0.10	–0.52	[–1.31, 0.26]
**Age in years**	**0.22**		**0.06**			**[0.02, 0.11]**	**0.18**	**0.04**	**[0.05, 0.07]**
**BPD severity**	–0.03	–0.04	[–0.22, 0.15]	–0.01	–0.01	[–0.15, 0.13]			
**Depression severity**	0.06	0.03	[–0.05, 0.11]	0.06	0.02	[–0.04, 0.08]			
**BPD vs. MOPD diagnosis**	–0.11	–0.64	[–1.59, 0.31]	–0.09	–0.38	[–1.09, 0.33]			
**Regression 4 (stepwise)**									
**Age in years**	**0.25**	**0.07**	**[0.03, 0.11]**	**0.18**	**0.04**	**[0.01, 0.06]**			

Bold numbers are significant on a *p* = .05 level

*BPD* Borderline personality disorder, *MOPD* Mixed and other personality disorders, *ToM* Theory of Mind, *CI 95%* 95% confidence interval

Correct Tables 3 and 4 are shown below:

**Table 3:** Regressions of diagnosis, BPD severity, depression severity, sex, and age on ToM total correct responses and exceeding ToM errors

Predictors	ToM total			exceeding ToM		
	β	*B*	CI 95%	β	*B*	CI 95%
**Sex** (0 = female, 1 = male)	–0.03	–0.37	[–2.49, 1.74]	**0.16**	**1.31**	**[0.00, 2.62]**
**Age in years**	**–0.23**	**–0.13**	**[–0.22, –0.05]**	0.00	0.00	[–0.05, 0.05]
**BPD severity**	–0.10	–0.25	[–0.59, 0.08]	**0.16**	**0.23**	**[0.02, 0.44]**
**BPD vs. MOPD diagnosis**	0.10	1.18	[–0.74, 3.11]	0.06	0.43	[–0.76, 1.61]
**Regression 2 (enter)**						
**Sex** (0 = female, 1 = male)	–0.02	–0.22	[–2.35, 1.90]	**0.17**	**1.39**	**[0.09, 2.68]**
**Age in years**	**–0.21**	**–0.12**	**[–0.21, –0.04]**	–0.01	–0.00	[–0.06, 0.05]
**Depression severity**	**–0.14**	**–0.15**	**[–0.29, -0.01]**	**0.18**	**0.12**	**[0.03, 0.21]**
**BPD vs. MOPD diagnosis**	0.08	0.95	[–0.96, 2.86]	0.09	0.65	[–0.51, 1.81]
**Regression 3 (enter)**						
**Sex** (0 = female, 1 = male)	–0.02	–0.34	[–2.45, 1.77]	0.15	1.29	[–0.01, 2.59]
**Age in years**	–0.22	–0.12	[–0.21, –0.04]	–0.02	–0.01	[–0.06, 0.05]
**BPD severity**	–0.05	–0.13	[–0.49, 0.24]	0.10	0.15	[–0.07, 0.38]
**Depression severity**	–0.13	–0.14	[–0.29, 0.02]	0.13	0.08	[–0.01, 0.18]
**BPD vs. MOPD diagnosis**	0.10	1.28	[–0.64, 3.20]	0.05	0.37	[–0.82, 1.55]
**Regression 4 (stepwise)**						
**Age in years**	**–0.26**	**–0.15**	**[–0.23, –0.07]**	not included		
**Depression severity**	**–0.13**	**–0.14**	**[–0.28, –0.003]**	**0.17**	**0.11**	**[0.02, 0.20]**

Bold numbers are significant on a *p* = .05 level

*BPD* Borderline personality disorder, *MOPD* Mixed and other personality disorders, *ToM* Theory of Mind, *CI 95%* 95% confidence interval

**Table 4:** Regressions of diagnosis, BPD severity, depression severity, sex, and age on reduced and no ToM errors

Predictors	Reduced ToM			No ToM		
	β	*B*	CI 95%	β	*B*	CI 95%
Regression 1 (enter)						
**Sex** (0 = female, 1 = male)	-0.06	-0.38	[-1.42, 0.67]	-0.10	-0.52	[-1.30, 0.26]
**Age in years**	**0.23**	**0.07**	**[0.02, 0.11]**	**0.18**	**0.04**	**[0.01, 0.07]**
**BPD severity**	-0.01	-0.01	[-0.17, 0.16]	0.01	0.01	[-0.11, 0.14]
**BPD vs. MOPD diagnosis**	-0.10	-0.62	[-1.57, 0.33]	-0.08	-0.37	[-1.08, 0.34]
**Regression 2 (enter)**						
**Sex** (0 = female, 1 = male)	-0.07	-0.46	[-1.50, 0.58]	-0.11	-0.61	[-1.44, 0.22]
**Age in years**	**0.22**	**0.06**	**[0.02, 0.10]**	**0.16**	**0.04**	**[0.00, 0.07]**
**Depression severity**	0.03	0.02	[-0.05, 0.09]	0.03	0.01	[-0.04, 0.07]
**BPD vs. MOPD diagnosis**	-0.11	-0.64	[-1.57, 0.30]	-0.06	-0.29	[-1.04, 0.45]
**Regression 3 (enter)**						
**Sex** (0 = female, 1 = male)	-0.06	-0.38	[-1.43, 0.66]	-0.10	-0.52	[-1.31, 0.26]
**Age in years**	**0.22**	**0.06**	**[0.02, 0.11]**	**0.18**	**0.04**	**[0.005, 0.07]**
**BPD severity**	-0.03	-0.04	[-0.22, 0.15]	-0.01	-0.01	[-0.15, 0.13]
**Depression severity**	0.06	0.03	[-0.05, 0.11]	0.06	0.02	[-0.04, 0.08]
**BPD vs. MOPD diagnosis**	-0.11	-0.64	[-1.59, 0.31]	-0.09	-0.38	[-1.09, 0.33]
**Regression 4 (stepwise)**						
**Age in years**	**0.25**	**0.07**	**[0.03, 0.11]**	**0.18**	**0.04**	**[0.01, 0.06]**

Bold numbers are significant on a *p* = .05 level

*BPD* Borderline personality disorder, *MOPD* Mixed and other personality disorders, *ToM* Theory of Mind, *CI 95%* 95% confidence interval

The original article was updated.
